# What Different Surveillance Systems Tell Us About Winter Respiratory Illness in England: A Time‐Series Analysis Combining Participatory, Syndromic and Laboratory Datasets, 2022–2025

**DOI:** 10.1111/irv.70323

**Published:** 2026-09-17

**Authors:** Katie Munro, Jonathon Mellor, Andre Charlett, Dan Todkill, Alex J. Elliot, Helen Hughes, Gavin Dabrera, SIREN Study Group, Sarah Foulkes, Victoria Hall, Anna Roynon, Maxine Nash, Stephen Winchester, Samuel Rowley, Clodagh Loughrey, Alison Grant, Rebecca Temple‐Purcell, Nick Wong, Ruth Penn, Eva McAlpine, Aran Dhillon, John Geen, Carla Pothecary, Eve Etell Kirby, Kim Gray, Elizabeth Boyd, Val Irvine, Tryphena Konala, Judith Radmore, Kim Hulacka, Sarah Knight, Davis Nwaka, Christian Hacon, Kathryn Hollinshead, Lois Bullen, Jacqueline Brandon, Kyra Holliday, Anu Chawla, Fran Westwell, Alexander Horsley, Shazaad Ahmad, Katherine Pagett, Sheron Clarke, Devesh Dhasmana, Susan Fowler, Euan Cameron, Anne Todd, Antonia Ho, Michael Murphy, Dawid Dytmer, Lita Kovina, Karen Black, Berni Welsh, Andrea Clarke, Jane Crowe, Martin Malcolm, Beth Smith, Ngozi Elumogo, Louise Coke, Sarah Brand, Jack Squires, Sumita Pai, Allison Doel, Esther Tarr, Charles Piercy, Sophia Strong‐Sheldrake, Vicky King, Fiona Shackley, James Pethick, Gareth Stephens, Simon Tazzyman, Sanal Jose, Jo Stickley, Alison Campbell, Susan Regan, Fiona Thompson, Angel Boulos, Tim Planche, Angela Houston, Liane Marsh, Barzo Faris, Brendan Payne, Jayne Harwood, Kathryn Court, Nikki White, Ruth Longfellow, Pauline Mercer, Matthew Halkes, Rajeka Lazarus, Aaran Sinclair, L. Berry, Frances Game, Christopher Holmes, Martin Wiselka, Karen Burns, Andrew Higham, Zehra’a Al‐Khafaji, Philippa Kemsley, Susan Hopkins, Victoria Hall, Jasmin Islam, Ana Atti, Omoyeni Adebiyi, Sophie Russell, Colin Brown, Sarah Foulkes, Jameel Khawam, Katie Munro, Angela Dunne, Irina Lut, Quisha Bustamante, Jesus Fresno, Nipunadi Hettiarachchi, Michelle Cairns, Declan Bradley, Kevin Wilson, Elen De Lacy

**Affiliations:** ^1^ Antimicrobial Resistance and Healthcare Associated Infections Division United Kingdom Health Security Agency (UKHSA) London UK; ^2^ Infectious Disease Modelling team United Kingdom Health Security Agency (UKHSA) London UK; ^3^ Real‐Time Syndromic Surveillance Team, Field Services, Health Protection in the Regions Chief Medical Advisor Group Birmingham UK; ^4^ Immunisation and Vaccine‐Preventable Diseases Division United Kingdom Health Security Agency (UKHSA) London UK; ^5^ SIREN Site Research Team UK; ^6^ UKHSA SIREN Team London UK; ^7^ Public Health Agency Northern Ireland Belfast UK; ^8^ Public Health Scotland Edinburgh UK; ^9^ Public Health Wales Cardiff UK

**Keywords:** epidemiology, influenza‐like illness, surveillance, winter pressures

## Abstract

**Background:**

A range of surveillance systems have been employed in England to monitor respiratory illness, including participatory, syndromic and laboratory datasets. Combined, they can provide powerful insights on the spectrum of respiratory illness across the population. We compared trends across these datasets over three successive winters.

**Methods:**

Participatory, syndromic and laboratory surveillance datasets from the UK Health Security Agency were integrated. Winter trends in influenza‐like illness (ILI), using a standardised definition, were described and compared across datasets, 2022/2023 to 2024/2025, using the cross‐correlation function (CCF). Multivariable (beta and negative binomial) regression models were used to explore the association between ILI and SARS‐CoV‐2, influenza and RSV positivity in each dataset.

**Results:**

Trends in ILI align well across participatory surveillance systems, with ILI peaking around Weeks 50–52 each season. Participatory surveillance system trends align well with syndromic healthcare use for ILI, with ILI symptoms peaking about a week before healthcare use. ILI in each dataset is driven by a different combination of respiratory viruses, with ILI in the community driven by a combination of SARS‐CoV‐2, influenza and RSV, whereas healthcare attendance was driven by influenza only.

**Conclusions:**

While each surveillance system differs in its purpose and population, trends in ILI align across datasets. Comparing a range of surveillance systems can provide increased confidence in each system. While syndromic healthcare use surveillance systems highlight the burden of severe illness, integration with participatory surveillance systems can demonstrate the wider burden across a spectrum of severity.

## 1 Introduction

Respiratory viruses, such as influenza, RSV and SARS‐CoV‐2, surge each winter in England, causing both illness in the community and increased pressure on health services [1, 2]. While these viruses exhibit seasonality, the timing and severity can differ each year, driven by changes in the circulating dominant influenza strains and SARS‐CoV‐2 variants. Surveillance of respiratory illness is therefore important to understand the impact in the community and on health services and, in turn, for planning mitigation strategies, such as targeting vaccination campaigns among more vulnerable groups. Maintaining vigilance around influenza and SARS‐CoV‐2 activity is particularly important due to the pandemic potential of each virus.

In order to understand the impact of respiratory illness, there are advantages to utilising a breadth of surveillance systems, including syndromic, participatory and laboratory surveillance. Syndromic surveillance systems collect and analyse health data in near real‐time and can provide an early warning system [3]. Participatory surveillance involves engaging members of the community to report on their health [4]. This complements syndromic surveillance, providing insights on the wider burden of disease, detecting less severe cases that do not present to healthcare services. Laboratory surveillance monitors respiratory viruses circulating in the population and can provide insights into which viruses may be driving illness and hospital attendances.

The UK Health Security Agency (UKHSA) respiratory programme coordinates a range of national systems across multiple domains, including syndromic, participatory and laboratory surveillance, to give a wide view of seasonal respiratory illness in England [5, 6]. Some of these are long‐standing, while several new surveillance systems were established during the COVID‐19 pandemic. Combined, this array of surveillance datasets can provide powerful insights on the spectrum of respiratory illness in the population, ranging from community cases to emergency department attendances. Each surveillance system is operated independently, and therefore, it is important to understand how these systems compare to one another.

This analysis aims to (1) compare trends in respiratory illness across participatory surveillance systems (healthcare workers (HCWs) and community) and to syndromic healthcare attendance datasets in England, (2) investigate associations between participatory/healthcare attendance surveillance and laboratory surveillance of positive SARS‐CoV‐2, influenza and RSV PCR results and (3) compare each surveillance system using the following metrics: timeliness, representativeness, flexibility, simplicity and acceptability.

## 2 Methods

### 2.1 Study Design

A time‐series analysis comparing participatory, syndromic and laboratory surveillance datasets.

### 2.2 Data Sources

SARS‐CoV‐2 Immunity and Reinfection Evaluation (SIREN): SIREN is a UK HCW cohort study established in response to the COVID‐19 pandemic with continuous data collection since June 2020 [7, 8]. Participants were employed at UK hospitals participating in the SIREN study and were aged 18 years and over. Participants complete fortnightly online surveys reporting acute symptoms experienced in the previous 2 weeks. Individual symptoms are selected by participants from a list provided (File S2), as well as onset date for each symptom.

Winter Coronavirus Infection Survey (WCIS): The Coronavirus Infection Survey (CIS) was established in response to the COVID‐19 pandemic and ran from April 2020 to March 2023 [9]. Participants included were from the residential population (people in fixed, noninstitutional housing, e.g., no care homes or prisons), aged 3 years and over. Following the completion of the CIS in March 2023, a separate Winter CIS (WCIS) was launched by the Office for National Statistics (ONS) and UKHSA in November 2023. Winter CIS participants were recruited from former CIS participants who had consented to be recontacted for future research studies [10]. WCIS participants completed monthly online surveys reporting symptoms experienced in the previous 7 days, from a list provided and the earliest onset date [10].

FluSurvey: FluSurvey is a long‐standing participatory surveillance system, established in 2009 in the wake of the A(H1N1) influenza pandemic. It is an online survey‐based surveillance system, running each winter, with participants from the general UK population aged 18 years and over [11]. Participants complete weekly surveys in which they report on the presence of symptoms experienced in the previous 7 days or since their last survey, from a list provided [12].

Emergency Department Syndromic Surveillance System (EDSSS): EDSSS captures daily anonymised patient attendance information from patients of all ages across Type 1 emergency departments (EDs) in England [13, 14]. The EDSSS routinely reports on attendances grouped into syndromic indicators, such as “acute respiratory infection” and “gastroenteritis”. Indicators are identified from the Systematized Nomenclature of Medicine Clinical Terms (SNOMED‐CT) codes recorded as a primary clinical diagnosis in each attendance record. These diagnoses are not laboratory confirmed.

UKHSA Remote Health Advice Syndromic Surveillance System: The daily number of phone calls triaged by the National Health Service (NHS) 111 service in England is captured [13]. UKHSA monitors anonymised NHS 111 triaged call data each day, including patients of all ages, covering the whole population of England. Syndromic indicators (such as “cold/flu”, “diarrhoea” or “sore throat”) are grouped based on triaged NHS 111 call outcomes classified using the NHS Pathways clinical system [15].

Respiratory DataMart: Respiratory DataMart is a sentinel surveillance system that captures laboratory testing results for all major respiratory viruses in England, with 17 laboratories reporting real‐time polymerase chain reaction (PCR) results weekly [16].

### 2.3 Definitions

Influenza‐like illness (ILI) was defined using the European Centre for Disease Prevention and Control (ECDC) definition (at least one of: fever, headache, malaise, myalgia and at least one of: cough, sore throat or shortness of breath) [17]. This was applied to the data provided by SIREN, WCIS and FluSurvey.

ED attendances in EDSSS are categorised into indicators, using the primary diagnosis entered into the patient record by ED clinicians, based on clinical judgement and patient management. The EDSSS ILI indicator uses 40 SNOMED‐CT diagnosis codes.

For the UKHSA Remote Health Advice Syndromic Surveillance System, the NHS 111 service uses a symptom‐based approach to triage callers. Those callers reporting cold or influenza symptoms are recorded within the Pathways system with an endpoint of “cold or flu”, which is used for the “cold/flu” syndromic surveillance indicator.

### 2.4 Variables

The proportion of surveys that reported ILI symptoms was calculated by ILI onset week for SIREN and FluSurvey. As symptom onset week was not available for FluSurvey, a proxy onset week was calculated as report week minus 7 days. The number of surveys reporting ILI symptoms was calculated by ILI onset week for WCIS.

The daily number of ED attendances for ILI (EDSSS) and the daily number of triaged calls for cold/flu (NHS 111) were aggregated by week.

For Respiratory DataMart data, SARS‐CoV‐2, influenza and RSV PCR positivity were aggregated by sample collection week.

### 2.5 Inclusion

Data were available from September to March, 2022/2023 to 2024/2025, for all datasets, except WCIS (November to March 2023/2024 only). Data were restricted to 18 years and over for WCIS and Respiratory DataMart, and to 15 years and over for EDSSS and NHS 111, to be able to compare to SIREN. FluSurvey data were provided for those aged 55 years and over, and therefore, each dataset was also restricted to older age groups for the comparisons to FluSurvey (SIREN and WCIS: 55 years and over. EDSSS and NHS 111: 65 years and over).

### 2.6 Descriptive Analysis

Three‐week moving averages were calculated for each season and dataset. Moving averages were then standardised to produce *z*‐scores, to compare datasets on the same scale. Raw data, smoothed 3‐week averages and *z*‐scores were presented for each dataset, overall and by age group (SIREN and WCIS: 18–54 and 55 years and over, EDSSS and NHS 111: 15–64 and 65 years and over).

### 2.7 Statistical Analysis

The cross‐correlation function (CCF) was used to quantify the correlation between datasets at time lags of up to ±3 weeks. CCFs were calculated for each season separately. A 5% significance threshold was calculated as *1.96/ √n*, where *n* is the length of the time series, for each comparison.

Regression models were used to estimate the association between ILI and SARS‐CoV‐2, influenza and RSV positivity across all three seasons for each dataset. Separate models were built with SARS‐CoV‐2, influenza or RSV positivity as the independent variables. Additional models were built with all three viruses as independent variables. Each model was adjusted for season.

For datasets with proportions (SIREN, FluSurvey), a beta regression model was used with a logit link. For each observation *i*, the outcome *p*_*i*_ was assumed to follow a Beta distribution (*μ*_*i*_ *ϕ, (1 − μ*_*i*_ *)ϕ*), where *μ*_*i*_ denotes the mean and *ϕ* is a precision parameter. The mean was related to the covariates through the following:

*logit (μ_i_) = β_0_ + β_1_ covid_i_ + β_2_ flu_i_ + β_3_ rsv_i_ + γ_1_ I (season_i_ = 2023/2024) + γ_2_ I (season_i_ = 2024/2025).*

Adjusted odds ratios (aORs) and 95% confidence intervals (CIs) were presented.

For datasets with counts (WCIS, EDSSS, NHS 111), a negative binomial regression model was used with a log link to account for overdispersion. For each observation *i,* the outcome *Y*_*i*_ was modelled as *Y*_*i*_ *~ NegBin* (*μ*_*i*_ *, κ)*, where *μ*_*i*_ is the expected count and *κ* is a dispersion parameter. The expected value was modelled as follows:

*log (μ_i_) = α_0_ + α_1_ covid_i_ + α_2_ flu_i_ + α_3_ rsv_i_ + δ_1_ I (season_i_ = 2023/2024) + δ_2_ I (season_i_ = 2024/2025).*

Adjusted incident rate ratios (aIRRs) and 95% CIs were presented.

The number of participants completing surveys for SIREN, WCIS and FluSurvey did vary each week, but completion rates were fairly consistent through the analysis period. Only 1 week of surveillance data was unavailable within one dataset. Three‐week moving averages were used for CCF and regression analysis, thereby reducing the impact of this missingness. CCF analyses were restricted to weeks for which data were available in both datasets being compared. No formal imputation of missing data was undertaken.

Statistical analysis was conducted in R v4.4.1. Additional packages used for analysis were *slider* for calculating moving averages, *effectsize* for standardising data, *betareg* for the beta regression models and *MASS* for the negative binomial regression models.

### 2.8 Summarising Each Surveillance System

Each surveillance system was summarised based on selected key metrics [18]. The purpose of each surveillance system was described, as well as frequency of data collection, reporting timeliness, participants included, simplicity, acceptability and flexibility of each system.

## 3 Results

### 3.1 Comparing Trends in ILI Across Participatory Surveillance Datasets in the Community and HCWs

**FIGURE 1:**
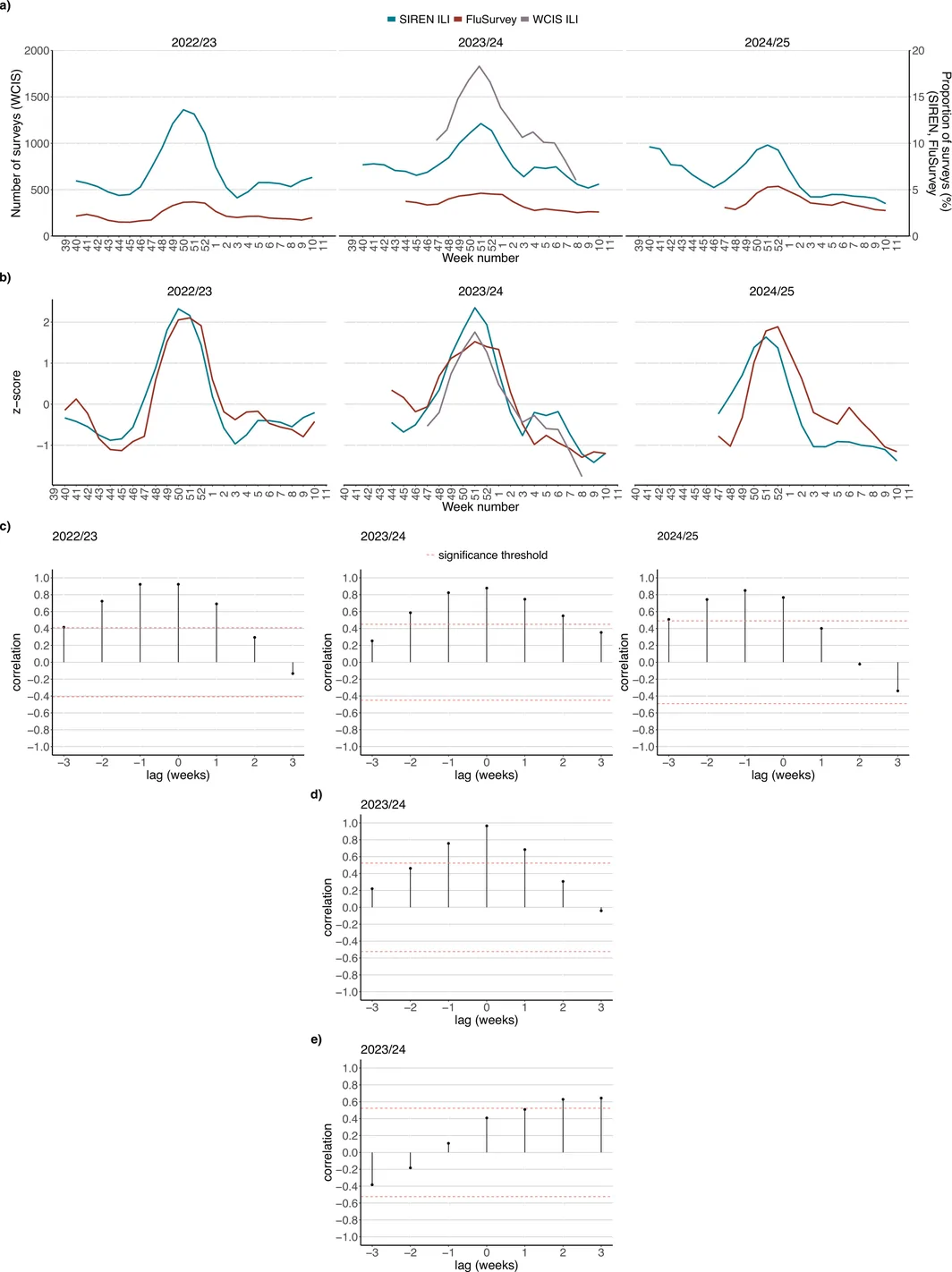
Number (WCIS) and proportion (SIREN, FluSurvey) of participants, aged 55 years and over, reporting influenza‐like illness (ILI) symptoms by symptom onset week, 2022/2023 to 2024/2025. (a) Trend in ILI and cold/flu calls by 3‐week moving averages. (b) Standardised trend in ILI and cold/flu calls by 3‐week moving averages. (c) Cross‐correlation of the trend in ILI (SIREN HCWs and FluSurvey community) by lag in weeks. (d) Cross‐correlation of the trend in ILI symptoms (SIREN HCWs and WCIS community) by lag in weeks. (e) Cross‐correlation of the trend in ILI symptoms (FluSurvey community and WCIS community surveillance) by lag in weeks. Statistically significant correlations are points crossing the 95% confidence limits (red dotted line). Time lags between trends are calculated in weeks. For example, 1 week time lag difference would be represented by the peak at lag ±1, that is, one lag week difference between the two trends.

Temporal trends in ILI were similar each season between HCWs and community participants, with ILI symptoms peaking in Weeks 50–52 each winter. The proportion of participants reporting ILI symptoms was consistently higher among HCWs each season (peak each season, SIREN HCWs: 18.0%, 13.2% and 13.5%), compared to community participants (FluSurvey community: 4.4%, 5.0% and 6.0%) (Figure S1a,b). Trends in ILI among HCWs and community participants correlate well each winter (SIREN HCWs vs. FluSurvey community: 55 years and over—maximum CCF 0.85–0.92 at 0‐ to 1‐week lead. SIREN HCWs vs. WCIS community: 55 years and over—maximum CCF 0.96 at 0 week, and 18–54 years—maximum CCF 0.99 at 0 week) (Figures 1 and S2).

### 3.2 Comparing Trends in Respiratory Illness From Participatory Surveillance Datasets to Syndromic Healthcare Attendance Datasets

#### 3.2.1 ILI in HCWs (SIREN), ED Attendances for ILI and NHS 111 Triaged Calls for Cold/Flu

**FIGURE 2:**
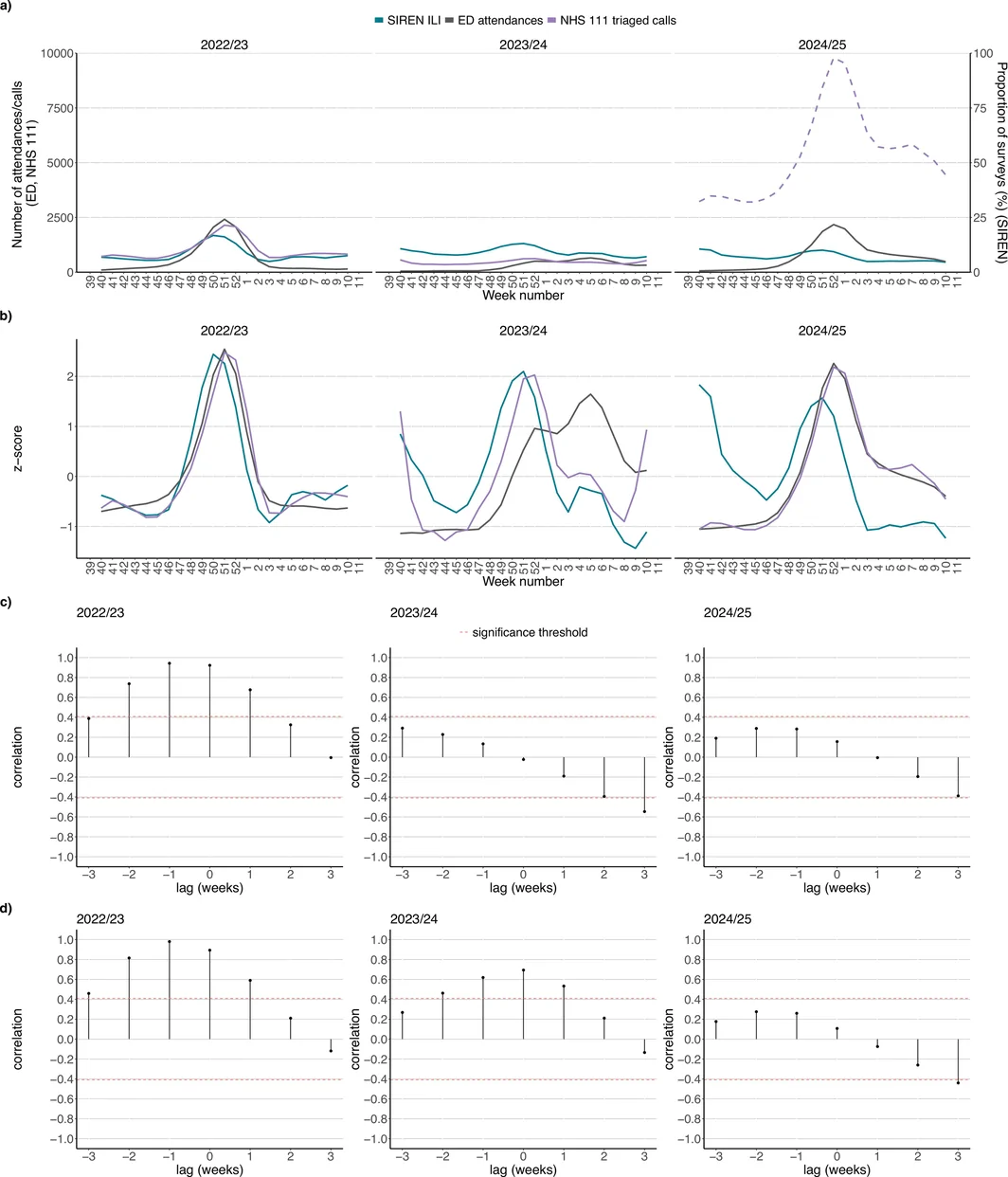
Proportion of participants reporting influenza‐like illness (ILI) in SIREN, number of ED attendances for ILI (ages 15 and over) and number of NHS 111 triaged calls for cold/flu (ages 15 and over), by week, 2022/2023 to 2024/2025. (a) Trend in ILI and cold/flu calls by 3‐week moving averages. (b) Standardised trend in ILI and cold/flu calls by 3‐week moving averages. (c) Cross‐correlation of the trend in ILI (SIREN HCWs and ED attendances) by lag in weeks. (d) Cross‐correlation of the trend in ILI (SIREN HCWs) and cold/flu calls (NHS 111) by lag in weeks. Dashed line: There were changes to data processing for the NHS 111 data during 2024/2025; further details in Table 2.

Trends in ED attendances for ILI and NHS 111 triaged calls for cold/flu followed a similar trend each season, peaking at weeks 51–52. However, in 2023/2024, a second peak (Weeks 4–5) and a lower number of ED attendances were observed, and in 2024/2025, the number of NHS 111 triaged calls was much higher than previous years (Figures 2a and S1d,e). Trends in HCW ILI (SIREN) correlated well with trends in ED attendances for ILI and NHS 111 triaged calls for cold/flu in 2022/2023, with HCW ILI symptom onset peaking about 1 week before ED attendances and NHS 111 triaged calls (SIREN HCWs vs. ED attendances: maximum CCF 0.92–0.94 at 0‐ to 1‐week lead, SIREN HCWs vs. NHS 111: 0.98 at 1‐week lead). SIREN HCW ILI followed a similar trend to NHS 111 triaged calls for cold/flu in 2023/2024 (maximum CCF 0.69 at 0 weeks lag). However, no clear correlation was observed between SIREN HCW ILI and NHS 111 triaged calls for cold/flu in 2024/2025 or between SIREN HCW ILI and ED attendances for ILI in 2023/2024 and 2024/2025 (Figure 2).

The proportion of participants reporting ILI in the HCW cohort (SIREN) was highest among those aged 18–54 years, compared to those aged 55 years and over. Similarly, the number of ED attendances for ILI and NHS 111 triaged calls for cold/flu was also higher among younger age groups (15–64 years vs. 65 years and over) (Figure S3).

#### 3.2.2 Community ILI (FluSurvey), ED Attendances for ILI and NHS 111 Triaged Calls for Cold/Flu

**FIGURE 3:**
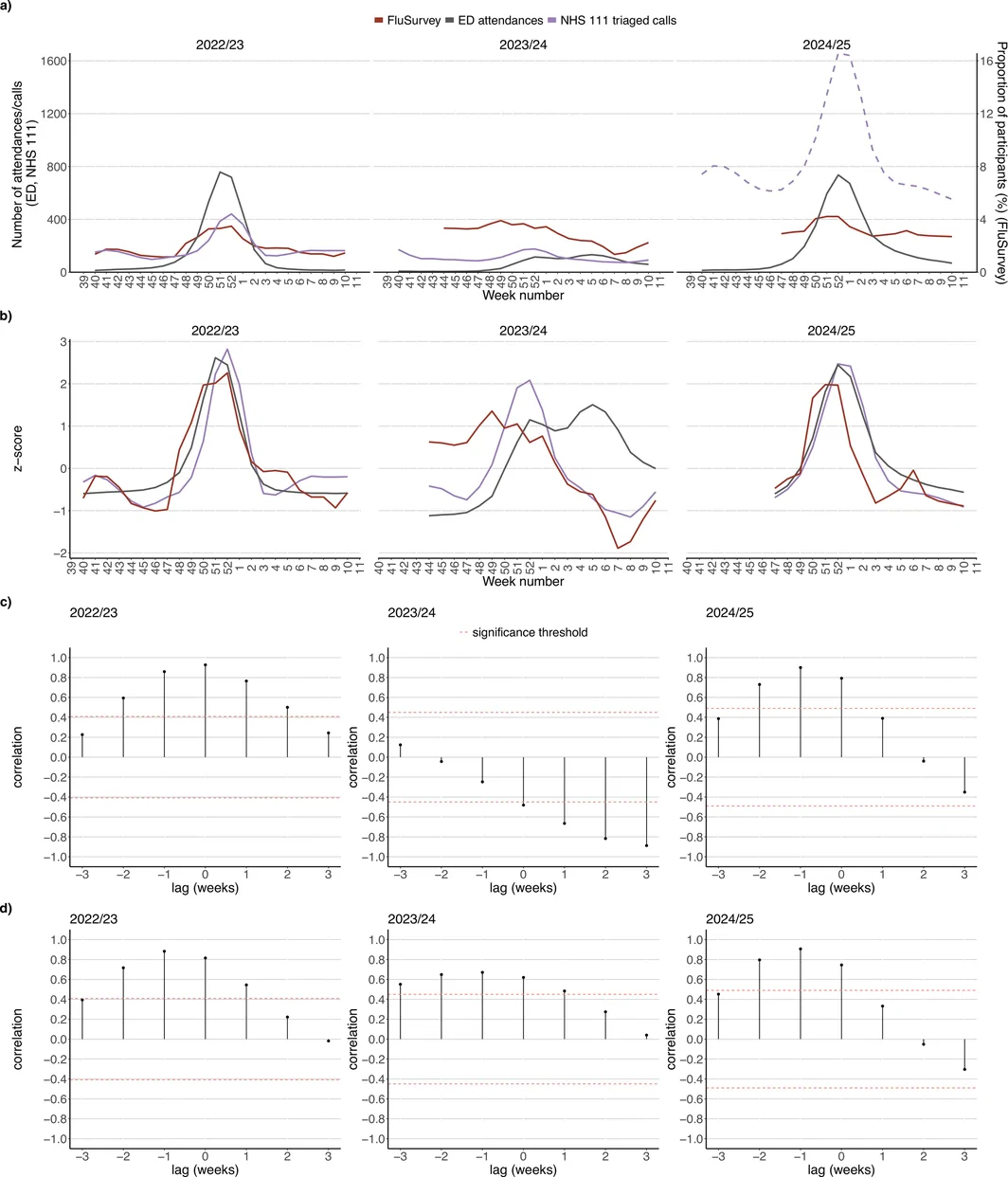
Proportion of participants reporting Influenza‐like illness (ILI) in FluSurvey, number ED attendances for ILI and number of NHS 111 triaged calls for cold/flu, ages 65 and over, by week, 2022/2023 to 2024/2025. (a) Trend in ILI and cold/flu calls by 3‐week moving averages. (b) Standardised trend in ILI and cold/flu calls by 3‐week moving averages. (c) Cross‐correlation of the trend in ILI (FluSurvey community and ED attendance) by lag in weeks. (d) Cross‐correlation of the trend in ILI (FluSurvey community) and cold/flu calls (NHS 111) by lag in weeks. Dashed line: There were changes to data processing for the NHS 111 data during 2024/2025; further details in Table 2.

Among participants aged 65 years and over, ILI reports in the community (FluSurvey) followed a similar trend to ED attendances for ILI and NHS 111 triaged calls for cold/flu in 2022/2023 and 2024/2025 (FluSurvey community vs. ED attendances: maximum CCF 0.90–0.93 at 0‐ to 1‐week lead, FluSurvey community vs. NHS 111: maximum CCF 0.88–0.91 at 1 weeks lead). In 2023/2024, there was some evidence of a correlation between FluSurvey community ILI and NHS 111 triaged calls for cold/flu (maximum CCF 0.67 at 1 week lead), but no clear correlation between FluSurvey community ILI and ED attendances for ILI (Figure 3).

#### 3.2.3 Community ILI (WCIS), ED Attendances for ILI and NHS 111 Triaged Calls for Cold/Flu

In 2023/2024, the number of participants reporting ILI in the WCIS community cohort correlated well with the number of NHS 111 triaged calls for cold/flu (maximum CCF 0.82–0.86 at 0‐ to 1‐week lead) but did not appear to correlate with ED attendances for ILI (Figure S4).

### 3.3 Comparing Trends From Participatory Surveillance and Healthcare Attendance Datasets to Infection Trends From Testing Data

#### 3.3.1 Associations Between ILI Trends and SARS‐CoV‐2, Influenza and RSV Positivity

There was strong evidence for an association between ILI in SIREN HCWs and SARS‐CoV‐2 (aOR: 1.09, 95% CI: 1.07–1.11, *p* < 0.001), influenza (aOR: 1.03, 95% CI: 1.02–1.03, *p* < 0.001) and RSV positivity (aOR: 1.06, 95% CI: 1.03–1.08, *p* < 0.001) from the beta regression model, adjusting for all three viruses and season. Similarly, there was strong evidence for an association between ILI in the FluSurvey community cohort and all three viruses (SARS‐CoV‐2: aOR 1.05, 95% CI: 1.02–1.07, *p* < 0.001; influenza: aOR 1.02, 95% CI 1.01–1.02, *p* < 0.001; and RSV: aOR 1.04, 95% CI 1.02–1.07, *p* < 0.001).

Results from the negative binomial regression model, adjusting for all three viruses, indicate strong evidence for an association between ILI in the WCIS community cohort and influenza (aIRR: 1.06, 95% CI: 1.01–1.10, *p* = 0.007) and RSV positivity (aIRR: 1.16, 95% CI: 1.08–1.26, *p* < 0.001) in 2023/2024.

**FIGURE 4:**
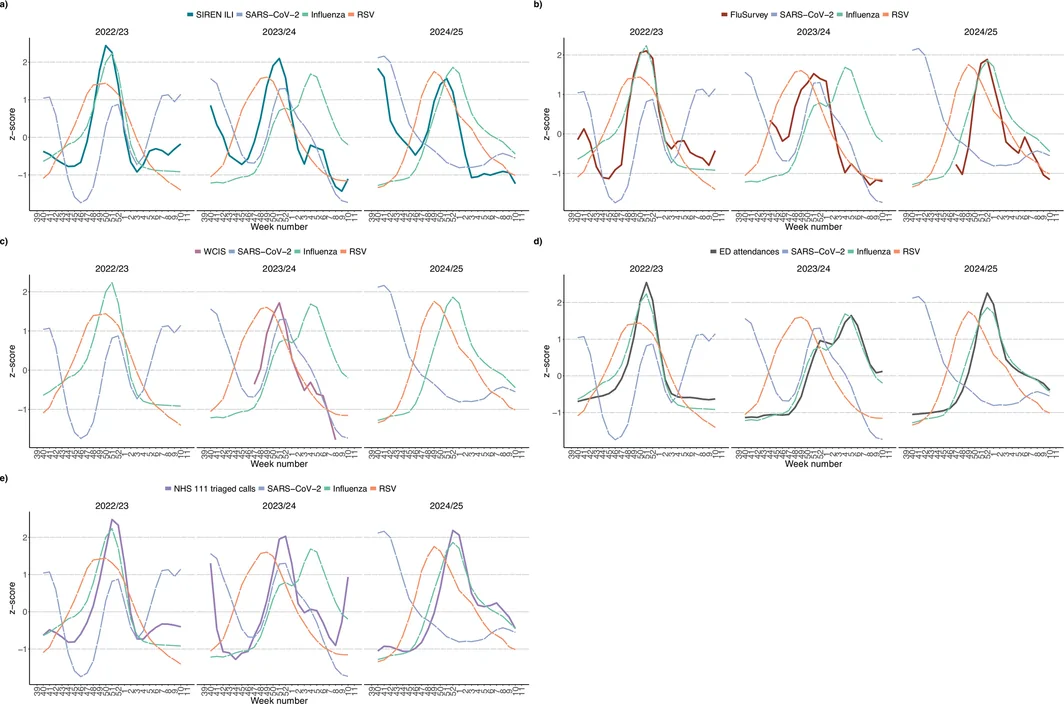
Standardised 3‐week moving averages of SARS‐CoV‐2, influenza and RSV positivity (ages 18 years and over). (a) Proportion of participants reporting influenza‐like illness (ILI), SIREN HCWs. (b) Proportion of participants reporting ILI, FluSurvey community (ages 55 years and over). (c) Number of participants reporting ILI, WCIS community. (d) Number of ED attendances for ILI (ages 15 years and over). (e) Number of NHS 111 triaged calls for cold/flu (ages 15 years and over).

After adjusting for all three viruses and season, there was strong evidence for an association between increased influenza positivity and both ED attendances for ILI and NHS 111 triaged calls for cold/flu (ED attendances: aIRR 1.14, 95% CI 1.13–1.16, *p* < 0.001; NHS 111: aIRR 1.04, 95% CI 1.03–1.05, *p* < 0.001) (Figure 4 and Table 1).

**TABLE 1:** Adjusted odds ratios (aORs)^a^ and incident rate ratios (aIRRs)^b^ for the association between SARS‐CoV‐2, influenza and RSV positivity and ILI in SIREN, FluSurvey and WCIS, ED attendances for ILI and NHS 111 triaged calls for cold/flu.

Variable	SIREN HCW ILI			FluSurvey community ILI			WCIS community ILI			ED attendances for ILI			NHS 111 triaged calls for cold/flu		
	aOR^a^	95% CI	*p*	aOR^a^	95% CI	*p*	aIRR^b^	95% CI	*p*	aIRR^b^	95% CI	*p*	aIRR^b^	95% CI	*p*
SARS‐CoV‐2 positivity	1.09	1.07–1.11	< 0.001	1.05	1.02–1.07	< 0.001	1.03	0.96–1.11	0.384	0.92	0.90–0.95	< 0.001	1.01	1.00–1.03	0.132
Influenza positivity	1.03	1.02–1.03	< 0.001	1.02	1.01–1.02	< 0.001	1.06	1.01–1.10	0.007	1.14	1.13–1.16	< 0.001	1.04	1.03–1.05	< 0.001
RSV positivity	1.06	1.03–1.08	< 0.001	1.04	1.02–1.07	< 0.001	1.16	1.08–1.26	< 0.001	0.95	0.91–0.99	0.021	0.99	0.96–1.01	0.369
Season															
2022/2023	—	—		—	—		—	—	—	—	—	—	—	—	—
2023/2024	1.24	1.11–1.38	< 0.001	1.63	1.45–1.84	< 0.001	—	—	—	0.95	0.78–1.15	0.618	0.58	0.51–0.65	< 0.001
2024/2025	1.12	0.99–1.26	0.069	1.90	1.58–2.28	< 0.001	—	—	—	0.93	0.75–1.16	0.490	5.41	4.76–6.14	< 0.001

^a^ Beta regression model shows the association between the proportion of ILI symptoms and the positivity of SARS‐CoV‐2, influenza and RSV, adjusting for the three winter seasons. Adjusted odds ratios (aOR) over one and statistical significance *p*‐value indicate a positive association. One percentage point increase in positivity leads to a ‘(aOR‐1) × 100’ multiplicative increase in the odds of the mean ILI proportion.

^b^ Negative binomial regression model shows the association between the number of ILI symptoms, ILI ED attendances and cold/flu calls and the positivity of SARS‐CoV‐2, influenza and RSV, adjusting for the three winter seasons. Adjusted incidence rate ratio (aIRR) over one and statistical significance *p*‐value indicate a positive association. One percentage point increase in positivity leads to a ‘(aIRR‐1) × 100’ multiplicative increase in the expected count.

### 3.4 Comparing Each Surveillance System by Timeliness, Representativeness, Simplicity, Acceptability and Flexibility

ED attendances, NHS 111 triaged calls and Respiratory DataMart surveillance systems receive data on a daily basis via an automated process, and surveillance outputs are reported on a weekly basis. All three of these systems collect data from the general population in England, across all ages, with ED and DataMart covering a geographical subset of the population. However, these three surveillance systems only capture cases that present to healthcare, which also generally reflect more severe cases.

SIREN, FluSurvey and WCIS are participatory surveillance systems and therefore require active participation from an engaged cohort, as well as study coordination. These surveillance systems also collect data less frequently than ED attendances, NHS 111 calls and Respiratory DataMart, receiving data on a weekly or fortnightly basis and do not capture all age groups. For example, SIREN and FluSurvey are restricted to adults aged 18 years and over, and the majority of participants in FluSurvey and WCIS are aged over 55 years. However, these participatory surveillance systems can provide a measure of respiratory illness in the community above those that present to healthcare (Table 2).

**TABLE 2:** A summary of each surveillance system (SIREN, FluSurvey, WCIS, ED attendances, NHS 111 triaged calls and Respiratory DataMart).

Dataset	Purpose	Frequency of data collection	Reporting timeliness (time between information being submitted to being reported)	Participants	Simplicity, acceptability and flexibility
SIREN [7]	Established during the COVID‐19 pandemic to monitor SARS‐CoV‐2 infections and immunity in HCWs. Expanded testing to influenza and RSV to understand the impact of winter pressures on the health service. Tracking trends in respiratory illness in HCWs since 2020.	Study participants complete fortnightly surveys reporting on symptoms experience in the previous 2 weeks. All participants are sent the survey on the same day.	Data received on a fortnightly basis and cover previous 2 weeks. One week delay between survey submission and reporting, to allow for participants to complete survey, data cleaning and analysis. Reporting trends to participants and UKHSA stakeholders.	UK HCWs aged 18 and over. Median age 45 [IQR 35–53] at enrolment; majority female and of white ethnicity.	Requires active participation from a closed cohort, and study team to coordinate data collection. Participants are re‐recruited each winter, high retention rates [19]. Flexibility to change survey questions each winter.
FluSurvey [11]	Monitoring trends in ILI in the general population. Forms part of InfluenzaNet, which monitors community ILI across Europe using a standardised survey [20].	Weekly surveys reporting on the previous 7 days. Participants are sent the survey on the same day each week.	Data received weekly and reported every week. Time from survey submission to reporting: Range 1–7 days ILI symptom rate and healthcare use reported in UKHSA weekly and annual influenza report [5, 6].	General population in England, with primary users aged 18 years and over. Mean age = 61 (2024/2025 season).	Requires active participation and study team coordination. New participants can enrol any time. Common questionnaire content across countries as part of InfluenzaNet.
WCIS [10]	Monitoring trends in SARS‐CoV‐2 infections and symptoms, to understand potential winter pressures on community/population.	Monthly surveys reporting on the previous 7 days. Data collection is staggered, with participants allocated to a 7‐day survey/testing wave, so data collected on a weekly basis.	Data received weekly, reported every 2 weeks. Time from survey submission to analysis: 1–7 days. Time from analysis to publication: + 7 days. Data reported on the gov.uk website [21].	General population in England and Scotland, aged 3 years and over. Majority (73.3%) aged 55 and over.	Requires active participation from a closed cohort, and study team coordination. Questionnaire designed by UKHSA and ONS.
ED attendances [13]	Monitoring trends in emergency department attendances, to provide an early warning of public health threats. Captures most severe cases that present to healthcare.	Daily	Data received daily and analysed, interpreted and reported internally within UKSA on a daily basis, to inform acute public health action. Summary data and key messages are reported in weekly UKHSA syndromic surveillance reports, and in weekly and annual UKHSA influenza reports [5, 6, 13].	General population in England, all ages included, from a network of 140 Type I ED sites (covering approximately 80% of Type I ED activity in England).	Emergency departments routinely record attendances electronically via an Emergency Department Information System (EDIS). Data are transmitted from EDISs to NHS England [22] and an anonymised subset is provided to the UKHSA ReSST via an automated feed. Diagnostic codes from EDISs are aggregated into syndromic indicators [23].
NHS 111 Calls [13]	Monitoring trends in phone calls triaged by the NHS 111 service, to monitor trends and provide an early warning of public health threats. Captures less severe cases that those accessing healthcare services in‐person.	Daily	Data received daily and analysed, interpreted and reported internally on a daily basis. Summary data and key messages are reported in weekly UKHSA syndromic surveillance reports, and in UKHSA weekly and annual influenza reports [5, 6, 13].	General population in England, all ages included.	NHS 111 call data are routinely recorded using the NHS Pathways clinical tool. Syndromic indicators are identified based on the main symptom for each call [24]. Anonymised call data are extracted to the UKHSA ReSST via an automated feed [24]. Up to week 192,023 UKHSA this feed was based on the NHS 111 Repeat Caller Service database. From week 202,023 onwards the feed was based on the NHS Pathways intelligent data tool. The
Respiratory DataMart [16]	Monitoring trends in respiratory viruses, from PCR testing at sentinel laboratories. Samples are predominantly provided from hospital patients, reflecting more severe cases that present to healthcare.	Daily	Data received daily and reported weekly. Data reported in UKHSA weekly and annual influenza reports [5, 6].	General population in England, all ages included, from 17 sentinel laboratories.	Respiratory virus test results are routinely recorded in laboratory information management systems, and data are extracted via an automated feed to the Respiratory DataMart System [25].

## 4 Discussion

Over the past three winter seasons, trends in ILI align well across participatory and syndromic systems in England, with subtle differences potentially due to differences in the respiratory viruses driving ILI in each dataset.

ILI symptom reporting from participatory surveillance systems followed a similar trend to the syndromic surveillance systems monitoring ED attendances for ILI and NHS 111 triaged calls for cold/flu. There was some evidence that ILI symptom reporting in HCWs and the community peaks about a week before reports of healthcare attendance. This finding likely reflects the real‐world situation, where symptomatic individuals wait for symptoms to resolve on their own and only contact healthcare if symptoms persist or worsen. This complements previous studies that have found ILI symptom trends peaking prior to hospital admissions [26].

There was strong evidence that all three viruses (SARS‐CoV‐2, influenza and RSV) were associated with ILI symptom reporting in participatory surveillance system, particularly SIREN and FluSurvey, whereas ED attendances for ILI and NHS 111 triaged calls for cold/flu were only associated with influenza positivity. This is likely due to the different severity profile and population covered by each dataset, with each virus also affecting a different population and presenting with a different severity profile. This highlights the importance of triangulation of datasets to understand the wider burden of respiratory illness over winter, suggesting that only using syndromic surveillance datasets would be less likely to capture the burden caused by viruses other than influenza.

Trends in infections from Respiratory DataMart can vary across seasons; for example, SARS‐CoV‐2 positivity follows a different trend each season, and trends in influenza positivity also differed slightly in each season. With each dataset impacted by a different combination of viruses, trends in ILI can also differ across seasons; for example, ED attendances had a notably different trend in 2023/2024. Additionally, the level of NHS 111 triaged calls for cold/flu was considerably higher in the 2024/2025 season. This increase was due to a NHS Pathways system update which updated the clinical triage of NHS 111 calls and had a significant impact on cold/flu (and other respiratory‐type) syndromic indicators. This type of change in working practices impacting on the data used for syndromic surveillance purposes is not unusual, and the resulting variability across seasons can make prediction more difficult.

The trends in ILI reported by HCWs mirrored those seen in the community cohorts each winter studied. However, rates of ILI were consistently higher in HCWs than community cohorts, with the peak each season being at least twice as high in SIREN than among FluSurvey participants (SIREN HCWs: 18.0%, 13.2% and 13.5%. FluSurvey community: 4.4%, 5.0% and 6.0%). This has been reported in other studies, for example, where higher rates of SARS‐CoV‐2 have been observed in HCWs compared to other occupations [27, 28, 29]. HCWs have been identified as a valuable population for surveillance for ongoing pandemic preparedness [30]. In addition, timely monitoring of HCW illness over winter can support workforce planning.

Higher rates of ILI were also seen in younger age groups (aged 18–54 years), in both symptom reporting and healthcare attendance. This highlights the need to consider age profile in participatory surveillance systems, which typically have higher representation of older participants (Table 2). As a working age population, the SIREN cohort at recruitment was younger, with a median age of 45, although has increased over time given participant ageing in a closed cohort. Consideration of how best to attract and retain younger individuals into longitudinal participatory surveillance systems is important for improving their representativeness.

The alignment between trends in ILI in each surveillance system provides increased confidence in each dataset. As summarised in Table 2, syndromic surveillance systems such as ED attendances and NHS 111 triaged calls are designed to monitor trends in near real‐time, with automation of data collection, and are therefore simple and timely surveillance systems. However, these syndromic datasets provide insight into cases of respiratory illness that present to healthcare and therefore reflect patients with symptoms that are of sufficient concern or severity for them to consult with a healthcare service/professional. Participatory surveillance systems require proactive engagement of community cohorts and can have less frequent data collection and a longer time from data collection to reporting. However, these participatory surveillance datasets are important to establish the burden of disease in the community beyond only those that present to healthcare. Maintaining an engaged cohort from participatory surveillance can also be utilised for multiple functions, such as conducting additional research through ad hoc questionnaires or collecting samples for testing. Triangulation of syndromic and participatory surveillance systems can therefore provide both an early warning system (from syndromic surveillance) and a wider picture of the burden of disease in the community across a range of severities, from mild symptoms in the community to more severe cases that present to healthcare.

The National Influenza and COVID‐19 surveillance reports and Influenza in the UK, annual epidemiological report from UKHSA, utilise data from multiple surveillance systems [5, 6]. However, while trends in all datasets are described and contribute to the key messages of the reports, at the moment, there is no comparison between the datasets presented. This highlights the importance of additional work to analyse multiple datasets together rather than independently. Other studies that have compared influenza or COVID‐19 surveillance systems have also highlighted the benefit of comparing multiple data sources rather than relying on a single system, particularly over multiple years [31, 32].

There are several limitations with this analysis. Data availability slightly differed between datasets; for example, WCIS data were only available for the 2023/2024 season, lacking the ability to compare trends for other years. Data for participants aged 55 years and over were only available for FluSurvey, restricting the age comparison between datasets. Additionally, 15 years and over data for ED attendances and NHS 111 triaged calls was compared to 18 years and over for SIREN, WCIS and DataMart data. Data were collected from UK cohorts for SIREN and FluSurvey, whereas data were only from England for the other datasets. However, the majority of participants in both SIREN and FluSurvey were from England. Symptom onset date was used to aggregate data for the SIREN and WCIS datasets, whereas a proxy symptom onset week was calculated for FluSurvey to compensate for the lack of symptom onset data. In addition, Respiratory DataMart samples are primarily from hospitalised patients, reflecting more severe cases, and therefore may be less comparable to ILI from participatory surveillance systems. The NHS Pathways system update in 2024/2025 greatly increased the counts of NHS 111 calls compared with previous seasons. However, the temporal pattern of calls remained similar to those observed in earlier seasons. Furthermore, the CCF analysis was conducted separately for each season using smoothed and standardised data, reducing the potential impact of differences in absolute call volumes between seasons. For the regression analysis, a sensitivity analysis excluding the 2024/2025 season was conducted. Although the overall findings were consistent, the estimated association with SARS‐CoV‐2 positivity was stronger when 2024/2025 was excluded, likely reflecting variability in SARS‐CoV‐2 positivity between seasons rather than the NHS Pathways system change. We therefore presented the model including all three seasons.

## 5 Conclusion

It is important to have a resilient surveillance system to monitor respiratory illness, with a breadth of coverage by population, virus and severity profile. Continued investment in a broad range of data systems is valuable for understanding the burden of respiratory illness, and integration is key to exploring the impact across a range of severities, while utilising the key strengths of each individual dataset.

## Author Contributions

**Katie Munro:** methodology, resources, data curation, validation, formal analysis, visualization, writing – original draft, writing – review and editing. **Jonathon Mellor:** methodology, validation, resources, data curation, writing – review and editing. **Andre Charlett:** methodology, writing – review and editing. **Dan Todkill:** resources, data curation, validation, writing – review and editing. **Alex J. Elliot:** validation, resources, data curation, writing – review and editing. **Helen Hughes:** validation, data curation, resources, writing – review and editing. **Gavin Dabrera:** validation, writing – review and editing, data curation, resources. **SIREN Study Group:** resources. **Sarah Foulkes:** methodology, supervision, writing – review and editing. **Victoria Hall:** conceptualization, writing – review and editing, supervision.

## Funding

A.J.E. receives funding from the National Institute for Health and Care Research (NIHR) Health Protection Research Unit (HPRU) in Gastrointestinal Infections at the University of East Anglia and the NIHR HPRU in Emergency Preparedness and Response at the University of Birmingham. A.J.E. and D.T. receive funding from the NIHR HPRU in Climate Change and Health Security at the London School of Hygiene and Tropical Medicine. Funding for the SIREN study was provided by the UK Health Security agency (UKHSA), the Department of Health and Social Care (DHSC) and contributions from the governments of Northern Ireland, Scotland and Wales. Additional funding was provided by the Medical Research Council (MRC) (MR/W02067X/1) and Health Data Research UK (HDR UK) (WP0034). FluSurvey is funded by the UK Health Security Agency. The views expressed in this article are those of the authors and not necessarily those of the NIHR, UKHSA or the DHSC.

## Ethics Statement

All participatory systems obtained informed consent from participants, which includes permission for secondary analyses of anonymised datasets by approved researchers. The SIREN study was approved by the Berkshire Research Ethics Committee (IRAS ID 284460, REC Reference 20SC0230) on 22 May 2020. Clinical trial registration number: ISRCTN11041050; registration date: 12 January 2021. The Winter Coronavirus Infection Study (WCIS) received ethical approval from the National Statistician's Data Ethics Advisory Committee. FluSurvey is a public health surveillance system; data are processed under the UK General Data Protection Regulation (UK GDPR) lawful bases that relate to public interest and public health: Article 6(1)(e) and Article 9(2)(i). UKHSA also has permissions under The Health Service (Control of Patient Information) Regulations 2002 (UK legislation), which allow for collection of data for public health purposes. Ethical approval was not necessary for the Emergency Department Syndromic Surveillance System, Remote Health Advice Syndromic Surveillance System or Respiratory DataMart. UKHSA has access to and presumptive authorisation to process and report in aggregate from a range of data sources under regulation 3 (Health Protection) of the Health Service (Control of Patient Information) Regulation 2002. Furthermore, the research in this study was carried out in compliance with the Helsinki Declaration.

## Conflicts of Interest

The authors declare no conflicts of interest.

## Supporting information


**Data S1:** List of SIREN study group members.

**Data S2:** Extract of SIREN study follow‐up questionnaire.

**Figure S1:** Weekly influenza‐like illness indicators, 2022/2023 to 2024/2025. (a) Weekly proportion of participants reporting ILI in SIREN, 2022/2023 to 2024/2025. (b) Weekly proportion of participants (aged 55 and over) reporting ILI in FluSurvey, 2022/2023 to 2024/2025. (c) Weekly number of participants (aged 18 and over) reporting ILI in WCIS, 2023/2024. (d) Weekly number of ED attendances for ILI in adults (aged 15 and over), 2022/2023 to 2024/2025. (e) Weekly number of NHS 111 triaged calls for cold/flu in adults (aged 15 and over), 2022/2023 to 2024/2025. (f) Weekly positivity of SARS‐CoV‐2, influenza and RSV in adults (aged 18 and over), Respiratory DataMart, 2022/2024 to 2024/2025.

**Figure S2:** Number of participants (aged 18 to 54 years) reporting ILI symptoms in SIREN and WCIS, by week, 2023/2024. (a) 3‐week moving averages. (b) Standardised 3‐week moving averages. (c) Cross‐correlation of ILI in SIREN HCWs and WCIS community.

**Figure S3:** Weekly influenza‐like illness indicators by age group, 2022/2023 to 2024/2025. (a) Weekly proportion of participants reporting ILI in SIREN, ages 18 to 54 years and 55 years and over, 2022/2023 to 2024/2025. (b) Weekly number of participants reporting ILI in WCIS, aged 18 to 54 years and 55 years and over, 2023/2024. (c) Weekly number of ED attendances for ILI in adults, ages 15 to 64 years and 65 years and over, 2022/2023 to 2024/2025. (d) Weekly number of NHS 111 triaged calls for cold/flu in adults, ages 15 to 64 years and 65 years and over, 2022/2023 to 2024/2025.

**Figure S4:** Number of participants reporting ILI in WCIS (ages 18 and over), number of ED attendances for ILI (ages 15 and over) and number of NHS 111 triaged calls for cold/flu (ages 15 and over), by week, 2023/2024. (a) 3‐week moving averages. (b) Standardised 3‐week moving averages. (c) Cross‐correlation of WCIS community ILI and ED attendances for ILI. (d) Cross‐correlation of WCIS community ILI and NHS 111 triaged calls for cold/flu.

## Data Availability

The data that support the findings of this study are available from the corresponding author upon reasonable request.
