# Supplementary material for: What Different Surveillance Systems Tell Us About Winter Respiratory Illness in England: A Time‐Series Analysis Combining Participatory, Syndromic and Laboratory Datasets, 2022–2025
Source: Influenza Other Respir Viruses. 2026 Sep 17;20(9):e70323. doi: 10.1111/irv.70323 (PMC13584328; doi:10.1111/irv.70323)
Supplement: Supplementary file 1 [file IRV-20-e70323-s004.pdf]

SIREN+ Follow-Up Questionnaire

Survey period: **{daterange}**

Symptoms

Between {date range}, have you started to experience any new symptoms in the following list?

Tick all that apply

- A new, continuous cough (coughing a lot for more than an hour, or 3 or more coughing episodes in 24 hours)
- Fever or high temperature (meaning you feel hot to touch on your chest or back)
- Shortness of breath
- Sore throat
- Blocked or runny nose
- Headache
- Muscle aches
- Joint pain
- Altered sense of smell
- Altered sense of taste
- Extreme fatigue
- Diarrhoea
- Nausea or vomiting
- Small, itchy red patches on fingers or toes
- Rash
- Swollen glands
- Feeling down or depressed
- Loss of concentration or brain fog
- Loss of appetite
- Other
    - Please specify [Free text]
- No, I have not got any new symptoms

For each symptom selected above,

When did these symptoms start and end?

Start date [Date picker]

End date [Date picker]
